# Investigating the Role of Hub Calcification Proteins in Atherosclerosis via Integrated Transcriptomics and Network-Based Approach

**DOI:** 10.3390/biology13110867

**Published:** 2024-10-25

**Authors:** Hajed Obaid A. Alharbi, Asifa Khan, Arshad Husain Rahmani

**Affiliations:** 1 Department of Medical Laboratories, College of Applied Medical Sciences, Qassim University, Buraydah 51452, Saudi Arabia; 2 Department of Molecular, Cell and Cancer Biology, UMass Chan Medical School, Worcester, MA 01605, USA; asifakhan0001@gmail.com

**Keywords:** atherosclerosis, cardiovascular disease, gene expression, feed-forward loop, weighted gene co-expression network, calcification

## Abstract

Atherosclerosis (AS) is a chronic inflammatory condition of the arteries, characterized by plaque formation that can restrict blood flow and lead to potentially fatal cardiovascular events. Given that AS is responsible for a quarter of global deaths, this study aimed to develop a systematic bioinformatics approach to identify biomarkers and regulatory targets involved in plaque development, with the goal of reducing cardiovascular disease risk. AS-specific mRNA expression profiles were retrieved from a publicly accessible database, followed by differentially expressed genes (DEGs) identification and AS-specific weighted gene co-expression network (WGCN) construction. Thereafter, calcification and atherosclerosis-specific (CASS) DEGs were utilized for protein–protein interaction network (PPIN) formation, followed by gene ontology (GO) term and pathway enrichment analyses. Lastly, AS-specific 3-node miRNA feed-forward loop (FFL) construction and analysis was performed. Microarray datasets GSE43292 and GSE28829 were obtained from gene expression omnibus (GEO). A total of 3785 and 6176 DEGs were obtained in case of GSE28829 and GSE43292; 3256 and 5962 module DEGs corresponding to GSE28829 and GSE43292 were obtained from WGCN. From a total of 54 vascular calcification (VC) genes, 20 and 29 CASS-DEGs corresponding to GSE28829 and GSE43292 were overlapped. As observed from FFL centrality measures, the highest-order subnetwork motif comprised one TF (*SOX7*), one miRNA (miR-484), and one mRNA (*SPARC*) in the case of GSE28829. Also, in the case of GSE43292, the highest-order subnetwork motif comprised one TF (*ESR2*), one miRNA (miR-214-3p), and one mRNA (*MEF2C*). These findings have important implications for developing new therapeutic strategies for AS. The identified TFs and miRNAs may serve as potential therapeutic targets for treating atherosclerotic plaques, offering insights into the molecular mechanisms underlying the pathogenesis and highlighting new avenues for research and treatment.

## Simple Summary

We procured publicly available mRNA expression profiles in cases of Atherosclerosis (AS) patients and obtained differentially expressed genes (DEGs) among these datasets. An AS-specific weighted gene co-expression network (WGCN) construction was established thereafter to identify hub modules. Then, calcification and atherosclerosis-specific (CASS) DEGs were utilized for protein-protein interaction network (PPIN) formation, followed by gene ontology (GO) term and pathway enrichment analyses. Lastly, an AS-specific 3-node miRNA feed-forward loop (FFL) construction and analysis was performed. This comprehensive approach improves our knowledge of the molecular landscape of AS patients and establishes a foundation for future treatment options and biomarkers, ultimately advancing precision therapeutics for AS and cardiovascular disease, thus paving the way for customized drugs and targeted treatments in the ongoing effort to improve patient outcomes.

## 1. Introduction

Atherosclerosis (AS) is a complex cardiovascular condition characterized by the buildup of plaque within the arterial walls (AWs), leading to narrowed and hardened arteries. This plaque is composed of calcium (Ca), cholesterol, fat, and other substances found in the blood. It is the most common cause of cardiovascular disease (CVD), accounting for half of all deaths in the Western world [1]. The disease progresses through the continuous development of AW lesions due to lipid retention (LR) in the intimal matrix. This retention causes arterial lumen stenosis (LS), which is a narrowing of the arterial lumen, and obstruction of blood flow. The progression can also lead to plaque rupture (PR). These processes can result in atherosclerotic thromboembolism, where a blood clot forms and can travel to other parts of the body, potentially causing a stroke or leading to peripheral artery disease (PAD) [2,3,4]. Another common condition is the presence of oxidized low-density lipoprotein (ox-LDL), which triggers an inflammatory response and recruits white blood cells (WBCs) to the AW. These cells differentiate into macrophages (MPs) that engulf the ox-LDL, becoming foam cells. Foam cells are a hallmark of early atherosclerotic lesions and contribute to the growth of the plaque [5]. In addition to this, calcification also plays a critical role in the development of advanced atherosclerotic lesions. This process involves the accumulation of Ca in the vascular tissues, which can be detected by computed tomography (CT) scans and serve as a subclinical marker of AS. The simultaneous occurrence of calcification in the coronary arteries and aortic valve calcification (AVC) supports the idea that AS often precedes these types of calcification. This suggests a close relationship between the progression of AS and the development of calcified lesions. Additionally, it is known that vascular smooth muscle cells (VSMCs) can transform into cells that resemble osteoblasts, which are responsible for bone formation. Once these advanced plaques have formed, they are irreversible, and currently, there are no medications available to treat or reverse this plaque formation [6].

The use of high-throughput sequencing (HTS) technology in recent years has been an effective tool in understanding the underlying genes and biological processes (BPs) during atherosclerotic plaque formation. Transcription factors (TFs) and microRNAs (miRNAs) are major in regulating gene expression. TFs are proteins that control the rate at which genes are transcribed into messenger RNA (mRNA) by binding to specialized deoxyribonucleic acid (DNA) sequences. The miRNAs are small RNA molecules that bind to mRNA and suppress or enhance protein synthesis. This complex interaction between miRNAs and TFs can either be inhibitory or synergistic. This synergy creates a feedback loop (FBL) that balances proper gene expression levels and regulation of TF and miRNAs. This relationship between miRNAs and TFs creates feed-forward loops (FFLs) or FBLs, in which TFs regulate miRNA, or miRNA inhibits the expression of TFs; both of them together coregulate the face of the standard target, which is a gene. Three types of FFLs are categorized based on the master regulators: TF-FFL, miRNA-FFL, and composite FFL [7].

The expression of genes is disrupted if this balance between TFs and miRNAs is dysregulated, as seen in many diseases, like CVD and cancer. Recently, many studies using bioinformatics and gene expression profiling (GEP) have found various genes and TFs. Several studies have highlighted the involvement of miRNAs and TFs in the regulation of gene expression related to AS and calcification. Specifically, miR-29a and miR-29b have been shown to prevent VSMCs calcification by suppressing the face of a disintegrin and metalloproteinase with thrombospondin motifs-7 (*ADAMTS-7*) [8]. In another study, they found that miR-125b downregulation can facilitate the calcification of VSMCs by targeting ETS proto-oncogene 1, transcription factor (*ETS1*), a TF [9]. RUNX family transcription factor 2 (*RUNX2*), a TF, is found to be the critical osteogenic regulator for osteoblast differentiation and chondrocyte maturation by repressing myocardin (*MYOCD*)-induced differentiation and promoting the calcification in plaque. MAF BZIP transcription factor F (*MAFF*) was found to regulate AS inflammation and cholesterol metabolism [10]. The studies were based on the results of studying the blood biomarkers in AS. However, GEP is more helpful in revealing the disease mechanism and its progression. Previous studies have shown that miRNAs and TFs may play a role in treating AS plaque formation and calcification; as the miRNAs and TFs have stability, specificity, and detectability, they can become excellent biomarkers. Therefore, identifying miRNA and TFs networks will take us one step closer to identifying the gene regulatory pathways involved in AS and potential therapeutic targets for plaque formation and calcification.

## 2. Materials and Methods

### 2.1. AS-Specific mRNA Expression Profile Selection and Differential Expression Analysis (DEA)

We accessed the National Center for Biotechnology Information (NCBI) gene expression omnibus (GEO) [11] (https://www.ncbi.nlm.nih.gov/geo/, accessed on 25 May 2024) to fetch mRNA expression profiles related to AS. All the search results were further streamlined in compliance with the inclusion criteria as follows: (i) all patients must be humans, and “expression profiling by array” must be the type of dataset(s); (ii) there must be availability of both preprocessed and raw data files for the dataset(s); (iii) there must be a minimum of 25 patient samples for the dataset(s); (iv) dataset(s) must comprise early versus advanced plaque tissue samples or carotid atheroma plaque versus normal carotid tissue samples from hypertensive population. Those studies lacking case reports, cell-line-based experimental study designs, non-homo sapiens samples, abstracts, and review articles were subject to exclusion. Pre-processed series matrix file of the finalized dataset(s) was downloaded. Sequential steps of batch correction, probe to HUGO gene nomenclature committee (HGNC) gene symbols mapping, and duplicate genes handling were performed as discussed previously [12]. All the batch-corrected expression values were z-score-transformed, followed by the elimination of 50% of low-variance (LV) genes. We then applied an unpaired two-sample *t*-test for computing ${log}_{2}(foldchange)$ and *p*-values of all genes via limma v3.60.6 R package [13]. Benjamini–Hochberg (BH) − *p*-value < 0.05 and $\left|{log}_{2}(foldchange)\right|>0.1$ was considered to be a significant threshold for screening differentially expressed genes (DEGs).

### 2.2. Non-Trait-Based AS-Specific Weighted Gene Co-Expression Network (WGCN) Construction and Analysis

The expression data of all AS-specific DEGs across patient samples were primarily given as an input to a Pigengene v1.30.0 R package [14] in order to discard any noisy DEGs. Then, we utilized all these non-noisy DEGs expression values across patient samples to identify and discard any sample outliers using a clustering-tree dendrogram. The sequential protocol of WGCN formation and module eigengene (ME) assignment was performed as discussed previously [12,15]. The ME dendrogram was investigated to check for any possible merging of modules with comparable high-expression trend patterns. We removed all modules comprising unnecessary or unassigned DEGs and retained the rest of the module DEGs for further analysis.

### 2.3. Protein–Protein Interaction Network (PPIN), Gene Ontology (GO) Term, and Pathway Enrichment Analyses

We accessed Harmonizome v3.0, a web-based tool [16] (https://maayanlab.cloud/Harmonizome/, accessed on 14 June 2024, to compile genesets pertaining to vascular calcification (VC). For this, we queried the Harmonizome web-based tool with the keyword ‘vascular calcification’ and downloaded all the respective genesets from multiple libraries. To maintain uniformity, we validated and retained all those unique VC genes which correlated with AS from literature studies. Next, AS-specific DEGs obtained from WGCN were overlapped with the VC genes (obtained from Harmonize) to obtain calcification and atherosclerosis-specific (CASS) DEGs. The CASS-DEGs were given as an input to a Search Tool for Retrieval of Interacting Genes (STRING) v12.0 database [17,18] (https://string-db.org/, accessed on 30 June 2024) for PPIN formation corresponding to a medium confidence (i.e., interaction score>0.4) and visualized via Cytoscape v3.10.1 [19]. The CASS-DEGs involved in PPIN were utilized as an input to Enrichr, a web-based tool [20,21] (https://maayanlab.cloud/Enrichr/, accessed on 12 July 2024), for compiling the top-ten most significant (*p*-value < 0.05) GO terms and pathway data. GO–molecular function (MF), GO–BP, GO–cellular compartment (CC), and Reactome libraries were utilized to compile GO terms and pathways data.

### 2.4. AS-Specific 3-Node miRNA FFL Construction and Analysis

All CASS-DEGs participating in PPIN were used for AS-specific 3-node miRNA FFL construction. The sequential protocol of compiling miRNA–mRNA, TF–mRNA, miRNA–TF pairs was performed as discussed previously [22]. All miRNAs and TFs were then validated via the literature, and those associated with AS and plaque formation were retained for further analysis. All interaction pairs (i.e., miRNA–mRNA, TF–mRNA, miRNA–TF) were unified together to construct an AS-specific 3-node miRNA FFL and subsequently visualized via Cytoscape.

## 3. Results

### 3.1. AS-Specific mRNA Expression Profile Selection and DEA

**Figure 1:**
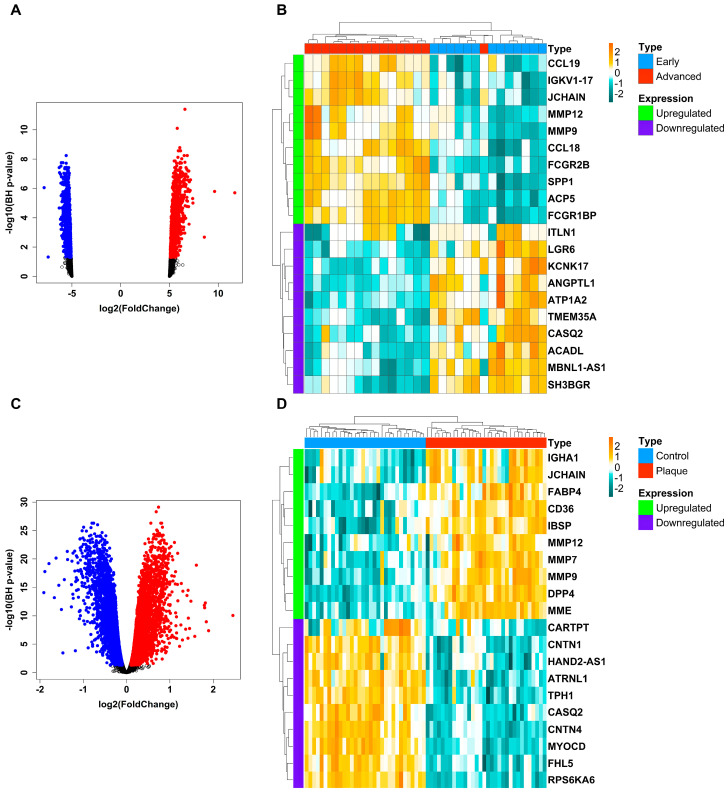
(**A**) The volcano plot showing the distribution of 3785 AS-specific DEGs (red dots specify upregulation, blue dots specify downregulation, and black dots specify non-significance) and (**B**) a heatmap showing the expression distribution of the top 10 upregulated, along with top 10 downregulated, AS-specific DEGs in the case of GSE28829. (**C**) The volcano plot showing the distribution of 6176 AS-specific DEGs (red dots specify upregulation, blue dots specify downregulation, and black dots specify non-significance) and (**D**) a heatmap showing the expression distribution of the top 10 upregulated, along with top 10 downregulated, AS-specific DEGs in the case of GSE43292. The vertical colored strip present at the left side of the heatmap signifies the up- and downregulation status of DEGs (i.e., magneta for downregulation and green for upregulation). Also, the horizontal colored strip present at the top side of the heatmap signifies the patient sample types.

Graphical abstract shows the detailed protocol highlighting our study objectives. Based on the aforementioned criteria, we selected two datasets with accession numbers GSE28829 and GSE43292. GSE28829 comprised 29 patient samples (16 advanced + 13 early atherosclerotic plaque from human carotid), while GSE43292 comprised 64 patient samples (32 carotid atheroma plaque + 32 normal carotid tissue). Post batch-correction, gene mapping, redundant, and 50% LV genes elimination, we were left with 9429 and 10,412 unique genes across GSE43292 and GSE28829 cohorts, respectively. A total of 3785 DEGs (upregulated: 2098+ downregulated: 1687) were obtained in the case of GSE28829, whereas 6176 DEGs (upregulated: 3114+ downregulated: 3062) were obtained in the case of GSE43292. Volcano plots in Figure 1A,B show the distribution of upregulated as well as downregulated DEGs along with nonsignificant genes in case of GSE28829 and GSE43292 cohorts. GTPase, IMAP family member 2 (*GIMAP2*) (BH − *p*-value $=4.03\times 10^{-12}$), and WDFY family member 4 (*WDFY4*) (BH − *p*-value $=7.50\times 10^{-30}$) were the most significant DEGs in the case of the GSE28829 and GSE43292 cohorts. Heatmaps in Figure 1C,D show the expression distribution of the top 10 upregulated and top 10 downregulated DEGs in the case of the GSE28829 and GSE43292 cohorts. Joining chain of multimeric IgA And IgM (*JCHAIN*) $[{log}_{2}(fold\;change)=2.68$] and ATPase ${Na}^{+}/K^{+}$ transporting subunit alpha 2 (*ATP1A2*) [${log}_{2}(fold\;change)=-1.65$] reported the top-fold change values across upregulated and downregulated DEGs in the case of GSE28829, while fatty-acid-binding protein 4 (*FABP4*) $[{log}_{2}(fold\;change)=2.45$] and contactin 1 (*CNTN1*) [${log}_{2}(fold\;change)=-1.91$] reported the top-fold change values across upregulated and downregulated DEGs in the case of GSE43292.

### 3.2. Non-Trait-Based AS-Specific WGCN Construction and Analysis

**Figure 2:**
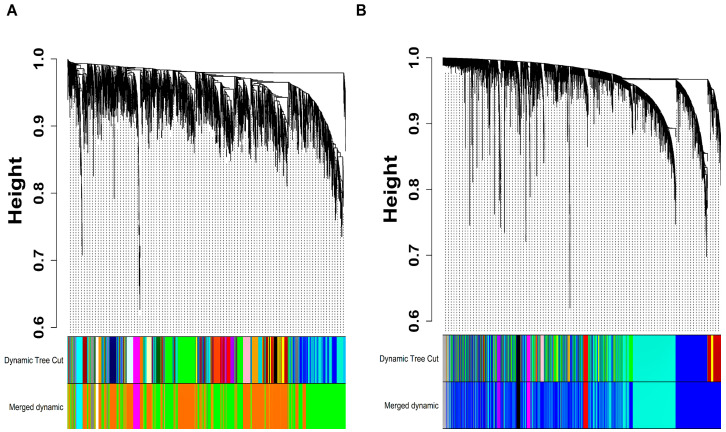

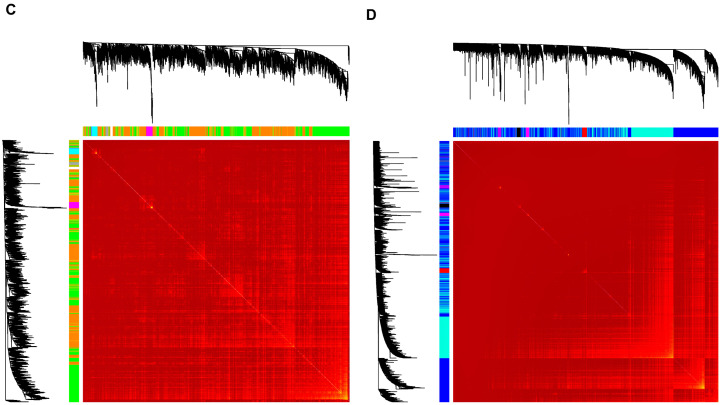
Clustering dendrograms (hierarchical) clustered based on dissTOM measured together with original and merged module colors in the case of (**A**) GSE28829 and (**B**) GSE43292. WGCN is represented as a TOM plot in the case of (**C**) GSE28829 and (**D**) GSE43292. Module assignments along with clustered gene dendrograms (hierarchical) are presented at the plot’s top and left side panels. Modules are signified by the dark-shaded blocks along the diagonal.

The expression data of 3257 and 6176 AS-specific DEGs were utilized as an input for WGCN establishment post noisy DEGs and sample outliers elimination corresponding to GSE28829 and GSE43292 datasets, respectively. The WGCNs were generated at β=10 (corresponding to $R^{2}=0.82$) and β=10 (corresponding to $R^{2}=0.8$) in the case of GSE28829 and GSE43292. Figures S1 and S2 show plots for β in consideration of scale-free topology (SFT) criteria corresponding to GSE28829 and GSE43292. The clustering dendrogram (hierarchical) and dynamic tree cut (DTC) algorithm resulted in 29 and 14 modules corresponding to GSE28829 and GSE43292. Tables S1 and S2 list the modules and their DEG counts in the case of GSE28829 and GSE43292. ME dendrograms were cut at a height of 0.25 equivalent to a correlation of 0.75 to merge highly co-expressed gene pattern modules in the case of GSE28829 and GSE43292. Post-merging, we obtained a total of 07 modules corresponding to GSE28829 and GSE43292 each (Figure 2A,B). Tables S3 and S4 list the modules and their DEG counts in the case of GSE28829 and GSE43292 post-merging. Since the gray module comprised unassigned DEGs, we eliminated it for further analysis. WGCN plots in Figure 2C,D capture the topological overlap matrix (TOM) information for all module genes post-merging in the case of GSE28829 and GSE43292. In total, 3256 and 5962 module DEGs corresponding to GSE28829 and GSE43292 were retained for further analysis.

### 3.3. PPIN, GO Term, and Pathway Enrichment Analyses

**Figure 3:**
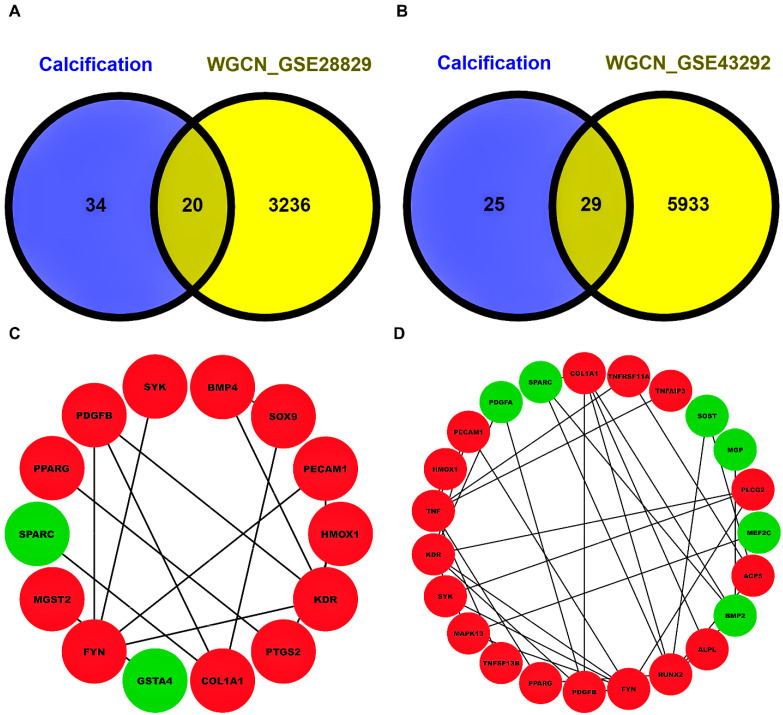
Venn plots showing overlapping 20 and 29 CASS-DEGs in the case of (**A**) GSE28829 and (**B**) GSE43292. Blue- and yellow-colored areas signify genesets corresponding to calcification and DEGs obtained from WGCN for both cohorts. Undirected and unweighted PPINs comprise 14 nodes interacting with 14 edges for (**C**) GSE28829, while 23 nodes interact with 35 edges for (**D**) GSE43292. Red- and green-colored nodes imply an upregulated and downregulated expression status of DEGs.

**Figure 4:**
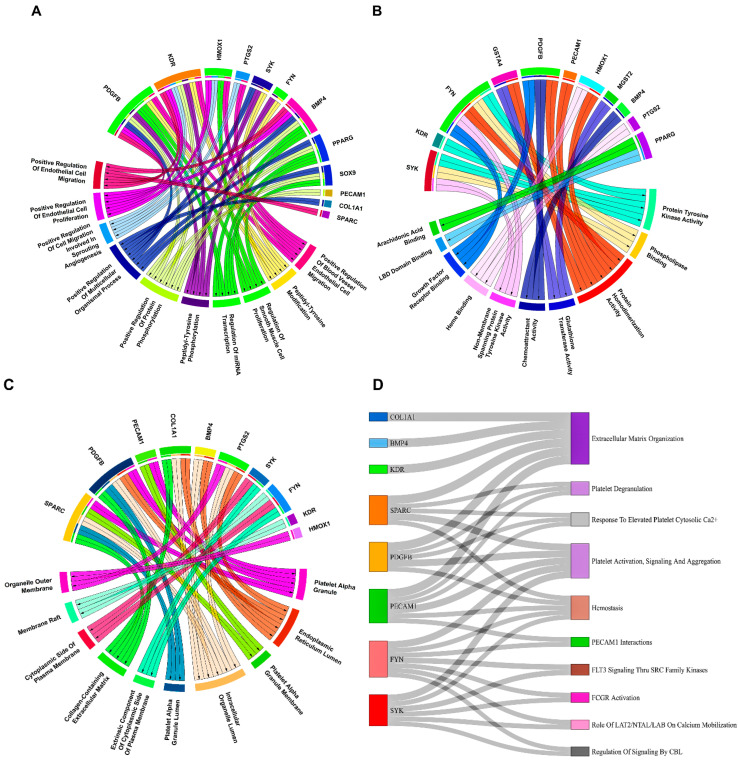
Linkage of top 10 significant (**A**) GO–BP, (**B**) GO–MF, (**C**) GO–CC terms (**D**) pathways with PPIN-participating CASS-DEGs corresponding to GSE28829 displayed by chord and sankey plots.

**Figure 5:**
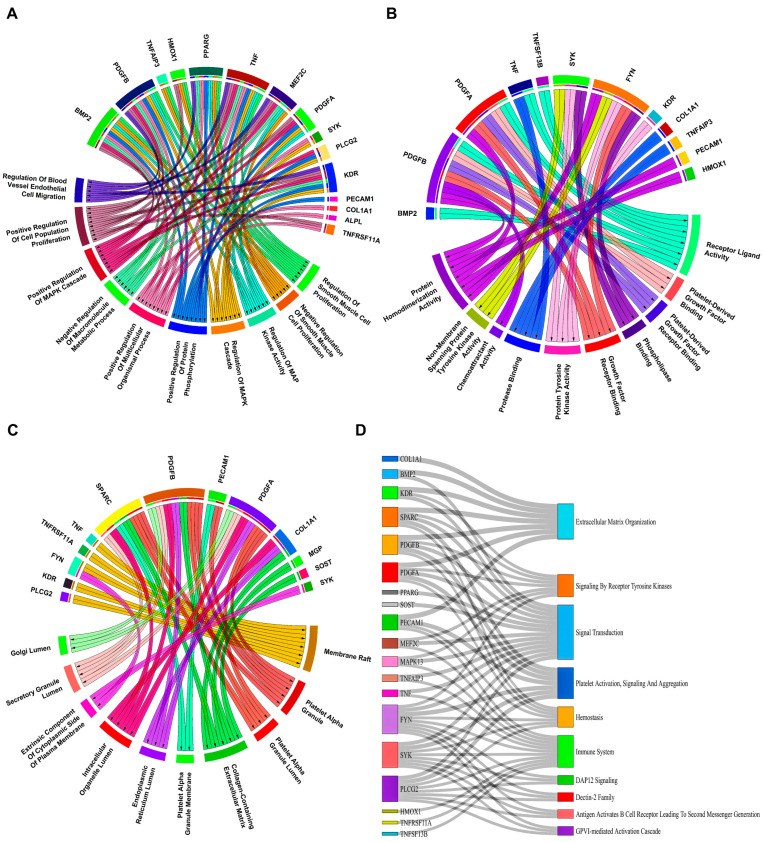
Linkage of top 10 significant (**A**) GO–BP, (**B**) GO–MF, (**C**) GO–CC terms (**D**) pathways with PPIN-participating CASS-DEGs corresponding to GSE43292 displayed by chord and sankey plots.

After careful evaluation of all VC-associated genesets from the Harmonize web-based tool and literature studies pertaining to AS, we compiled a total of 54 unique VC genes as shown in Table S5. Venn plots showing overlapping 20 and 29 CASS-DEGs corresponding to GSE28829 and GSE43292 cohorts are shown in Figure 3A,B. Undirected and unweighted PPINs, as shown in Figure 3C,D, comprised 14 nodes interacting with 14 edges for GSE28829 and 23 nodes interacting with 35 edges for GSE43292, respectively. Kinase insert domain receptor (*KDR*), FYN proto-oncogene, src family tyrosine kinase (*FYN*) [node degree(ND)=4], and *RUNX2* (ND=7) reported highest NDs in the case of GSE28829 and GSE43292. Associations of PPIN-participating CASS-DEGs with top 10 significant (i.e., *p*-value < 0.05) pathways, GO–BP, GO–MF, GO–CC terms corresponding to GSE28829 and GSE43292 were presented via sankey and chord plots, as shown in Figure 4 and Figure 5. A total of 8, 12, 11, and 10 PPIN-participating CASS-DEGs were involved in the top 10 significant pathways, GO–BP, GO–MF, GO–CC terms, corresponding to GSE28829. Meanwhile, a total of 19, 15, 12, and 13 PPIN-participating CASS-DEGs were involved in the top 10 significant pathways, GO–BP, GO–MF, GO–CC terms, corresponding to GSE43292. The most significant pathways, GO–BP, GO–MF, GO–CC terms, corresponding to GSE28829, were extracellular matrix (ECM) organization (*p*-value $=2.45\times 10^{-8}$), positive regulation of blood vessel endothelial cell migration (*p*-value $=2.41\times 10^{-8}$), protein tyrosine kinase activity (*p*-value $=4.12\times 10^{-5}$), and platelet alpha granule (*p*-value $=2.99\times 10^{-5}$). Meanwhile, the most significant pathways, GO–BP, GO–MF, GO–CC terms, corresponding to GSE43292, were ECM organization (*p*-value $=1.10\times 10^{-9}$), regulation of smooth muscle cell proliferation (*p*-value $=2.33\times 10^{-13}$), receptor ligand activity (*p*-value $=1.66\times 10^{-6}$), and membrane raft (*p*-value $=1.52\times 10^{-6}$).

### 3.4. AS-Specific 3-Node miRNA FFL Construction and Analysis

**Figure 6:**
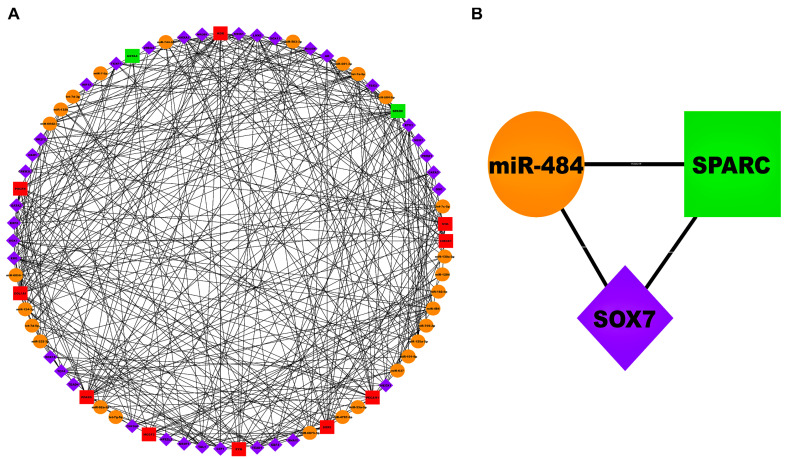
(**A**) Unweighted and undirected 3-node miRNA FFL comprising 74 nodes and 372 edges. (**B**) Highest-order subnetwork motif comprising one TF (*SOX7*), one miRNA (miR-484), and one mRNA (*SPARC*). Magenta-colored diamond nodes, orange-colored circular nodes, and green- and red-colored rectangular nodes signify TFs, miRNAs, and mRNAs, respectively.

**Figure 7:**
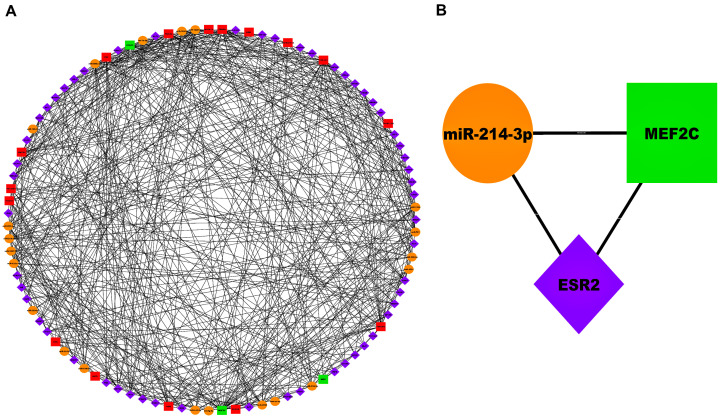
(**A**) Unweighted and undirected 3-node miRNA FFL comprising 95 nodes and 536 edges. (**B**) Highest-order subnetwork motif comprising one TF (*ESR2*), one miRNA (miR-214-3p), and one mRNA (*MEF2C*). Magenta-colored diamond nodes, orange-colored circular nodes, and green- and red colored-rectangular nodes signify TFs, miRNAs, and mRNAs, respectively.

Figure 6A and Figure 7A showed AS-specific 3-node miRNA-FFLs corresponding to GSE28829 and GSE43292 cohorts. FFL, as shown in Figure 6A, comprised 74 nodes and 372 edges. Within this FFL, ND of TFs, miRNAs, and mRNAs varied from 3 to 16, 3 to 17, and 5 to 27, respectively. Average NDs of TFs, miRNAs, and mRNAs were 8.74, 8.66, and 17, respectively. Meanwhile, the FFL shown in Figure 7A comprised 95 nodes and 536 edges. Within this FFL, ND of TFs, miRNAs, and mRNAs varied from 3 to 17, 3 to 29, and 6 to 30, respectively. The average NDs of TFs, miRNAs, and mRNAs were 8.69, 13.52, and 17, respectively. The breakup of FFL nodes and edges in case of GSE28829 and GSE43292 are summarized in Tables S6 and S7. Tables S8 and S9 show the top 3 TFs, miRNAs, and mRNAs ranked on the basis of betweenness, ND, and closeness in the case of GSE28829 and GSE43292. As observed from these centrality measures, the highest-order subnetwork motif comprised one TF [SRY-box transcription factor 7 (*SOX7*)], one miRNA (miR-484), and one mRNA [secreted protein acidic and cysteine rich (*SPARC*)] in the case of GSE28829. Also, in the case of GSE43292, the highest-order subnetwork motif comprised one TF [estrogen receptor 2 (*ESR2*)], one miRNA (miR-214-3p), and one mRNA [myocyte enhancer factor 2C (*MEF2C*)].

## 4. Discussion

This study aimed to describe the progression of AS from early to advanced stages by analyzing gene expression profiles from two datasets, namely GSE28829 and GSE43292, obtained from the GEO. These datasets are unique because they consist exclusively of samples from AS patients. Our study introduces a novel strategy for understanding AS progression using transcriptomics and a network-based approach.

The datasets include gene expression data from healthy individuals and AS patients across different age groups, including children, adults, and elders. In GSE28829, we obtained 3785 DEGs, with 2098 upregulated and 1687 downregulated. Whereas, in GSE43292, we obtained 6176 DEGs, with 3114 upregulated and 3062 downregulated.

AS is a complex disease involving genetic susceptibilities, metabolic disturbances, inflammatory processes, and hemodynamic factors. These higher number of DEGs observed may reflect the multifaceted nature of AS and its impact on different patient groups. In GSE28829, *JCHAIN* and *ATP1A2* showed the highest-fold change values among the upregulated and downregulated DEGs, respectively. *JCHAIN*, involved in immune response and Immunoglobulin A (IgA)/Immunoglobulin M (IgM) transport, influences inflammatory processes and may play a role in AS pathogenesis. *ATP1A2*, encoding the α−2 isoform of ${Na}^{+}/K^{+}$ ATPase, is essential for maintaining ${Na}^{+}$ and $K^{+}$ ion gradients, and its dysregulation could contribute to VSMC abnormalities in AS [23,24]. In GSE43292, *FABP4* was highly upregulated and *CNTN1* highly downregulated. *FABP4* is crucial in lipid metabolism and inflammatory responses, facilitating the formation of foam cells and promoting chronic inflammation within AWs [25,26]. *CNTN1* downregulation can enhance the expression of adhesion molecules like vascular cell adhesion molecule 1 (*VCAM-1*) and intercellular adhesion molecule 1 (*ICAM-1*), increasing leukocyte adhesion and local inflammation, which are critical in AS progression [27].

Key proteins with high-degree nodes in the PPINs, such as *KDR* and *FYN* in GSE28829 and *RUNX2* in GSE43292, were identified. *KDR* (*VEGFR2*) is involved in endothelial cell proliferation (CP) and migration, critical for angiogenesis and atherosclerotic plaque formation. *FYN*, a tyrosine kinase, plays a role in signal transduction pathways regulating cell growth and differentiation. *RUNX2* is crucial for VC and is correlated with AS progression.

Significant pathways identified in GSE28829 included ECM organization, endothelial cell migration, and protein tyrosine kinase activity. In GSE43292, key pathways involved smooth muscle cell proliferation, receptor–ligand activity, and membrane raft formation. These pathways highlight the BPs and molecular interactions underlying AS. The GO analysis identified potential PPINs and co-regulation associations in the GSE28829 cohort, including pathways such as ECM organization, platelet deregulation, response to elevated platelet cytosolic Ca, hemostasis, platelet and endothelial cell adhesion molecule 1 (*PECAM1*) interactions, Fms related receptor tyrosine kinase 3 (*FLT3*) signaling through SRC family kinases, Fc gamma receptor (FCGR) activation, linker for activation of T cells 2 (*LAT2*)/non-T-cell-activation linker (*NTAL*)/linker for activation of B cells *(LAB*) role in Ca mobilization, and regulation of signaling by Cbl Proto-Oncogene (*CBL*). For the GSE43292 cohort, significant pathways included ECM organization, signaling by receptor tyrosine kinases, signal transduction, platelet activation, signaling and aggregation, hemostasis, immune system responses, transmembrane immune signaling adaptor TYROBP (*DAP12*) signaling, the Dectin-2 family, antigen activation of the B-cell receptor leading to second messenger generation, and glycoprotein VI (GPVI)-mediated activation cascade.

Our study utilized a regulatory network-based approach to detect specific mRNAs, miRNAs, and TFs forming an AS-specific closed 3-node miRNA FFL. Analyzing these FFLs can enhance understanding of the cell and molecular pathways dysregulated in AS, thus elucidating its pathophysiology. This study identified the top 3 TFs, miRNAs, and mRNAs ranked by betweenness, ND, and closeness centrality in both GSE28829 and GSE43292 datasets.

So, our first hypothesis revolves around the highest-order subnetwork motif, including one TF (*SOX7*), one miRNA (miR-484), and one mRNA (*SPARC*) in GSE28829. miR-484 serves as a molecular marker for the development of carotid plaques and their susceptibility to rupture. It demonstrated significant enrichment in both diseased endothelial progenitor cells (PCs) and plasma from individuals with coronary heart disease (CHD). Thus, it is plausible to suggest that miR-484 is crucial for maintaining vascular endothelial cell (VEC) homeostasis and preventing endothelial dysfunction [28]. Emerging evidence suggests that *SOX7* may play a role in modulating inflammatory responses and VC, both of which are critical in the pathogenesis of AS. Inflammatory cytokines and calcification are key drivers of plaque instability and rupture, leading to clinical events such as heart attacks and strokes [29,30]. *SPARC* and *SOX7* both play critical roles in AS by regulating endothelial function, VSMC behavior, and inflammatory responses [30,31]. *SPARC* is involved in ECM remodeling and cell–matrix interactions, while *SOX7* influences gene expression related to endothelial integrity and inflammation [30,32]. Their coordinated activity impacts plaque stability, angiogenesis, and overall vascular health, contributing to the progression of AS. It is plausible that their interaction could exacerbate atherosclerotic processes. miR-484 might influence *SOX7* expression or activity, and *SOX7* might influence the *SPARC* expression or activity, thereby affecting the downstream pathways involved in endothelial dysfunction and VC. Understanding this interaction could provide insights into new therapeutic targets for preventing or treating AS.

Another hypothesis revolves around the connection of TF (*ESR2*), miRNA (miR-214-3p), and mRNA (*MEF2C*). Both *ESR2* and *MEF2C* are involved in regulating endothelial and VSMC functions. *ESR2* signaling can influence the activity of *MEF2C*, thereby modulating the expression of genes critical for vascular health [33,34,35]. Conversely, *MEF2C* is a direct target of miR-214-3p. Downregulation of *MEF2C* by miR-214-3p can impact VSMC differentiation and endothelial function, contributing to atherosclerotic processes [35,36]. *ESR2* mediates the protective effects of estrogens against cardiovascular diseases by reducing the expression of inflammatory cytokines and adhesion molecules, thus maintaining vascular homeostasis and preventing AS progression [33,34,37]. The overexpression of miR-214-3p has been shown to suppress autophagy in human umbilical vein endothelial cells (HUVECs) when these cells are exposed to ox-LDL, a key factor in AS. This suppression leads to increased ox-LDL accumulation, which exacerbates endothelial dysfunction and promotes the adhesion of monocytes (Mo), contributing to the inflammatory processes in AS. miR-214-3p serves as a crucial molecular link between impaired autophagy and endothelial cell dysfunction in AS. By targeting and downregulating essential autophagy-related proteins, miR-214-3p exacerbates the damage to endothelial cells, thereby promoting the progression of AS. This understanding highlights the potential of miR-214-3p as a therapeutic target for preventing or mitigating endothelial dysfunction and AS [38].

Based on the above hypotheses, we can conclude that miR-484 might regulate *SOX7* and *SPARC*, while *SOX7* could regulate *SPARC* expression. Additionally, miR-214-3p negatively regulates *MEF2C* expression, whereas *ESR2* signaling positively influences *MEF2C* expression. To validate these hypothesis, further in vitro studies are required. Understanding the interplay of these mechanisms is crucial for unraveling AS complexities and developing effective prevention strategies and targeted treatments. Ongoing research strives to decipher molecular underpinnings, identify novel biomarkers, and devise personalized approaches to combat AS and its cardiovascular complications. In this study, we explored the multifaceted landscape of AS, examining its pathogenesis, risk factors, clinical manifestations, diagnostic modalities, therapeutic interventions, and the evolving paradigm of precision medicine in cardiovascular care.

## Data Availability

The expression datasets pertaining to GSE28829 and GSE43292 were downloaded from NCBI-GEO available at https://www.ncbi.nlm.nih.gov/geo/query/acc.cgi?acc=GSE28829, assessed on 25 May 2024 and https://www.ncbi.nlm.nih.gov/geo/query/acc.cgi?acc=GSE43292, assessed on 25 May 2024.

## Supplementary Materials

The following supporting information can be downloaded at: https://www.mdpi.com/article/10.3390/biology13110867/s1, Figure S1: Plots for choosing appropriate value of β in case of GSE28829; Figure S2: Plots for choosing appropriate value of β in case of GSE43292; Table S1: List of original modules and their gene count in case of GSE28829; Table S2: List of original modules and their gene count in case of GSE43292; Table S3: List of merged modules and their gene count in case of GSE28829; Table S4: List of merged modules and their gene count in case of GSE43292; Table S5: List of vascular calcification geneset; Table S6: Relationship between regulatory elements in case of GSE28829 FFL; Table S7: Relationship between regulatory elements in case of GSE43292 FFL; Table S8: Top 3 regulatory elements in case of GSE28829 FFL; Table S9: Top 3 regulatory elements in case of GSE43292 FFL.
