# Supplementary material for: Investigating the Role of Hub Calcification Proteins in Atherosclerosis via Integrated Transcriptomics and Network-Based Approach
Source: Biology (Basel). 2024 Oct 25;13(11):867. doi: 10.3390/biology13110867 (PMC11592380; doi:10.3390/biology13110867)
Supplement: Supplementary file 1 [file biology-13-00867-s001.zip › biology-3263223-supplementary.pdf]

Supporting Data

Investigating the role of hub calcification proteins in Atherosclerosis via integrated transcriptomics and network-based approach

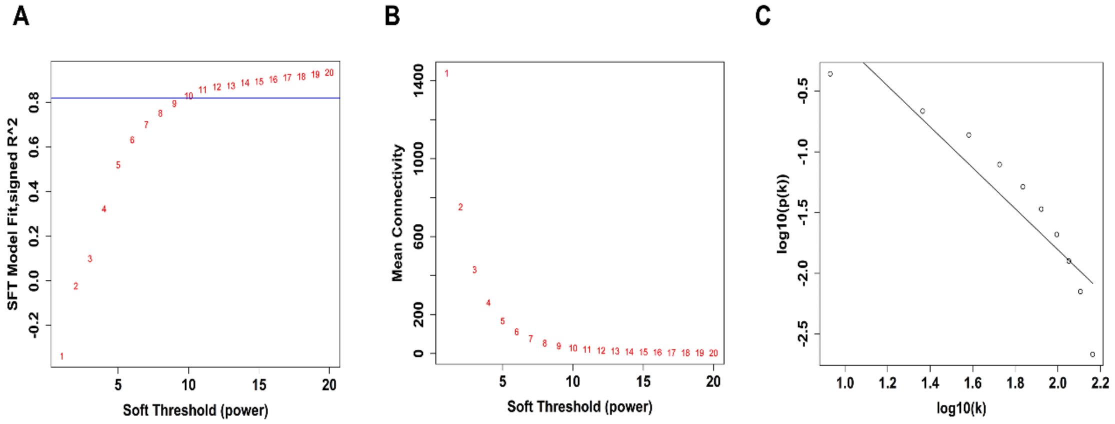

Figure S1. ***(A)*** *Analysis of scale-free fitting indices* (R^2) *for various possible soft-thresholding powers* (β)*.* ***(B)*** *Analysis of mean connectivity for various possible* β *values.* ***(C)*** $log_{10}(k)$ *vs* $log_{10}(p(k))$ *plot where the scale-free topology is depicted by the approximate straight-line relationship (high* $R^2 = 0.82$*) and a negative value of slope* $(slope = -1.69)$ *when* $\beta = 10$.

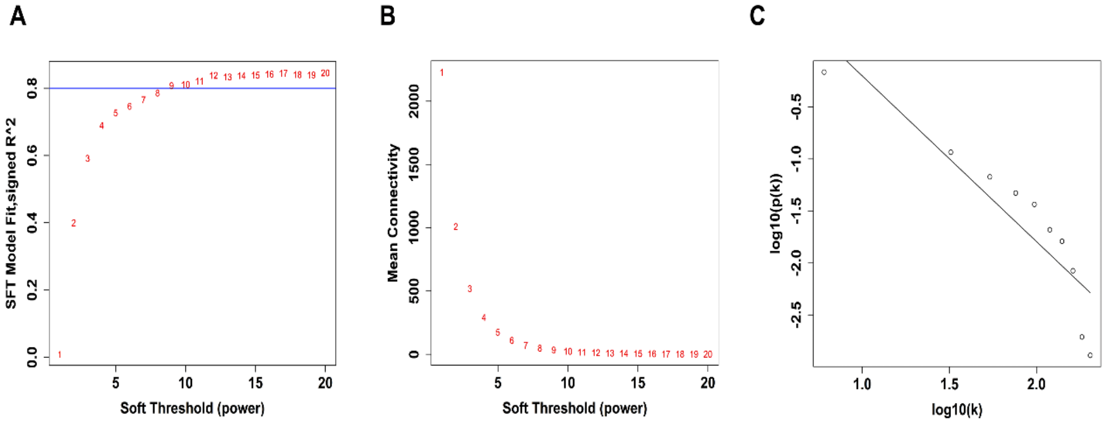

Figure S2. ***(A)*** *Analysis of scale-free fitting indices* (R^2) *for various possible soft-thresholding powers* (β). ***(B)*** *Analysis of mean connectivity for various possible* β *values.* ***(C)*** $log_{10}(k)$ *vs* $log_{10}(p(k))$ *plot where the scale-free topology is depicted by the approximate straight-line relationship (high* $R^2 = 0.8$*) and a negative value of slope* ($slope = -1.59$) *when* $\beta = 10$.

Table S1. *Original modules (with color chromatic legends) and their DEGs count for GSE28829.*

Module		No. of DEGs	Module		No. of DEGs
**Blue**		298	**Greenyellow**		86
**Black**		116	**Grey**		01
**Cyan**		78	**Grey60**		71
**Brown**		255	**Lightcyan**		77
**Darkgreen**		48	**Lightgreen**		70
**Darkgrey**		35	**Lightyellow**		59
**Darkorange**		34	**Magenta**		96
**Darkred**		53	**Midnightblue**		78
**Darkturquoise**		46	**Orange**		34
**Green**		198	**Pink**		97
**Purple**		87	**Royalblue**		54
**Red**		119	**Salmon**		79
**Skyblue**		31	**Turquoise**		732
**Tan**		81	**White**		32
**Yellow**		212			

Table S2. *Original modules (with color chromatic legends) and their DEGs count for GSE43292.*

Module	No. of DEGs	Module	No. of DEGs
**Blue**	1362	**Greenyellow**	50
**Black**	98	**Grey**	214
**Green**	326	**Pink**	85
**Brown**	659	**Tan**	43
**Purple**	80	**Turquoise**	2575
**Red**	145	**Yellow**	423
**Salmon**	35	**Magenta**	81

Table S3. *Merged modules (with color chromatic legends) and their DEGs count for GSE28829.*

Module	No. of DEGs	Module	No. of DEGs
**Darkgrey**	35	**Greenyellow**	1550
**Darkorange**	1465	**Grey**	01
**Cyan**	78	**White**	32
**Magenta**	96		

Table S4. *Merged modules (with color chromatic legends) and their DEGs count for GSE43292.*

Module		No. of DEGs	Module		No. of DEGs
**Black**		98	**Purple**		80
**Blue**		2983	**Grey**		214
**Turquoise**		2575	**Red**		145
**Magenta**		81			

Table S5. *Vascular calcification genelist.*

ACP5	AGG	ALK	ALPL
BMP2	BMP2K	BMP4	BSP
CABP4	CCDC85B	COL1A1	COL1A2
EDN1	EFCAB7	FOSL1	FYN
GRAP	GSTA3	GSTA4	GSTM6
GSTP2	HMOX1	IGSF1	KDR
LRCH2	MAPK12	MAPK13	MEF2C
MGP	MGST2	MSX2	NA
NRROS	OP	OPG	PDGFA
PDGFB	PECAM1	PLCG2	PPARG
PRKCZ	PTGS2	RUNX2	SOST
SOX9	SPARC	SRY	SYK
TNF	TNFAIP3	TNFRSF11A	TNFSF13B
TRPV4	TXNDC2		

Table S6. *Summary of relationships among mRNAs, miRNAs, TFs in case of GSE28829 FFL.*

Interaction	No. of edges	No. of miRNAs	No. of mRNAs	No. of TFs
miRNA-mRNA	66	27	12	-
miRNA-TF	168	27	-	35
TF-mRNA	138	-	12	35

Table S7. *Summary of relationships among mRNAs, miRNAs, TFs in case of GSE43292 FFL.*

Interaction	No. of edges	No. of miRNAs	No. of mRNAs	No. of TFs
miRNA-mRNA	58	21	18	-
miRNA-TF	226	21	-	55
TF-mRNA	252	-	18	55

Table S8. *Top 3 TFs/miRNAs/mRNAs ranked based on centrality measures such as degree, betweenness, and closeness in case of GSE28829 FFL.*

Regulatory Elements	Node Degree	Betweenness	Closeness
		**miRNA**	
miR-484	17	0.037	0.52
let-7e-5p	15	0.028	0.54
miR-6873-3p	13	0.015	0.48
		**TF**	
SOX7	15	0.036	0.54
ERG	15	0.020	0.50
TCF4	14	0.035	0.50
		**mRNA**	
SPARC	27	0.094	0.57
COL1A1	24	0.074	0.55
PPARG	22	0.065	0.53

Note: miRNAs, mRNAs, TFs highligjhted in red have the highest value of each centrality measure (i.e., Node degree, Betweenness, Closeness).

Table S9. *Top 3 TFs/miRNAs/mRNAs ranked based on centrality measures such as degree, betweenness, and closeness in case of GSE43292 FFL.*

Regulatory Elements	Node Degree	Betweenness	Closeness
**miRNA**			
miR-550a-3p	29	0.081	0.58
miR-214-3p	21	0.029	0.51
miR-1228-3p	21	0.036	0.52
**TF**			
TNFAIP3	26	0.052	0.52
ESR2	20	0.025	0.50
RUNX2	17	0.028	0.51
**mRNA**			
MEF2C	30	0.072	0.57
COL1A1	30	0.058	0.55
PPARG	29	0.049	0.52

Note: miRNAs, mRNAs, TFs highligjhted in red have the highest value of each centrality measure (i.e., Node degree, Betweenness, Closeness).
